# Surveillance for peri-elimination trachoma recrudescence: Exploratory studies in Ghana

**DOI:** 10.1371/journal.pntd.0009744

**Published:** 2021-09-20

**Authors:** Laura Senyonjo, James Addy, Diana L. Martin, David Agyemang, Dorothy Yeboah-Manu, Sarah Gwyn, Benjamin Marfo, Adwoa Asante-Poku, Agatha Aboe, Ernest Mensah, Anthony W. Solomon, Robin L. Bailey

**Affiliations:** 1 Research Team, Sightsavers, Haywards Heath, United Kingdom; 2 Clinical Research Department, London School of Hygiene & Tropical Medicine, London, United Kingdom; 3 Eye Health Department, Ghana Health Service, Accra, Ghana; 4 Division of Parasitic Diseases and Malaria, Centers for Disease Control and Prevention, Atlanta, United States of America; 5 Sightsavers Ghana, Accra, Ghana; 6 Bacteriology Department, Noguchi Memorial Institute for Medical Research, Accra, Ghana; 7 Neglected Tropical Diseases Division, Ghana Health Service, Accra, Ghana; 8 NTD team, FHI360, Accra, Ghana; RTI International, UNITED STATES

## Abstract

**Introduction:**

To date, eleven countries have been validated as having eliminated trachoma as a public health problem, including Ghana in 2018. Surveillance for recrudescence is needed both pre- and post-validation but evidence-based guidance on appropriate strategies is lacking. We explored two potential surveillance strategies in Ghana.

**Methodology/principal findings:**

Amongst randomly-selected communities enrolled in pre-validation on-going surveillance between 2011 and 2015, eight were identified as having had trachomatous-inflammation follicular (TF) prevalence ≥5% in children aged 1–9 years between 2012 and 2014. These eight were re-visited in 2015 and 2016 and neighbouring communities were also added (“TF trigger” investigations). Resident children aged 1–9 years were then examined for trachoma and had a conjunctival swab to test for *Chlamydia trachomatis (Ct)* and a dried blood spot (DBS) taken to test for anti-Pgp3 antibodies. These investigations identified at least one community with evidence of probable recent *Ct* ocular transmission. However, the approach likely lacks sufficient spatio-temporal power to be reliable.

A post-validation surveillance strategy was also evaluated, this reviewed the ocular *Ct* infection and anti-Pgp3 seroprevalence data from the TF trigger investigations and from the pre-validation surveillance surveys in 2015 and 2016. Three communities identified as having ocular *Ct* infection >0% and anti-Pgp3 seroprevalence ≥15.0% were identified, and along with three linked communities, were followed-up as part of the surveillance strategy. An additional three communities with a seroprevalence ≥25.0% but no *Ct* infection were also followed up (“antibody and infection trigger” investigations). DBS were taken from all residents aged ≥1 year and ocular swabs from all children aged 1–9 years. There was evidence of transmission in the group of communities visited in one district (Zabzugu-Tatale). There was no or little evidence of continued transmission in other districts, suggesting previous infection identified was transient or potentially not true ocular *Ct* infection.

**Conclusions/significance:**

There is evidence of heterogeneity in *Ct* transmission dynamics in northern Ghana, even 10 years after wide-scale MDA has stopped. There is added value in monitoring *Ct* infection and anti-*Ct* antibodies, using these indicators to interrogate past or present surveillance strategies. This can result in a deeper understanding of transmission dynamics and inform new post-validation surveillance strategies. Opportunities should be explored for integrating PCR and serological-based markers into surveys conducted in trachoma elimination settings.

## Author summary

The goal for trachoma programmes is elimination of trachoma as a public health problem. This means that ongoing low-level eye-to-eye transmission of the causative bacterium, *Chlamydia trachomatis* (*Ct*), is acceptable. Countries need to implement a suitable surveillance system to identify any return to higher transmission levels. The best methodology for doing this is not known. We first explored the approach used by Ghana in its standard programme, which involved monitoring a limited number of randomly selected communities for evidence of active (inflammatory) trachoma visible in children’s eyes on examination by trained observers. Although this strategy led to identification of at least one community that had probably had recent *Ct* transmission, the approach is unlikely to consistently identify places where return to higher levels of transmission is a risk. We also explored using information on infection (detected in eye swabs) and antibodies to *Ct* (detected in the blood) to identify communities at risk. We found evidence of both persistent eye-to-eye *Ct* transmission and areas where infection was transient and has now gone away. We conclude that the use of infection and antibody data for surveillance of trachoma appears promising.

## Introduction

Trachoma, caused by repeated conjunctival infection with the bacterium *Chlamydia trachomatis* (*Ct*), which can progress to trachomatous trichiasis (TT), the in-turning of the eyelids resulting in at least one eyelash touching the globe of the eye, is the leading infectious cause of blindness worldwide [1]. It is targeted for global elimination as a public health problem [2] using the “SAFE” strategy: Surgery to minimize progressive visual loss in individuals who have TT; mass drug administration [MDA] of Antibiotics; and Facial cleanliness and Environmental improvement. Elimination as a public health problem can be validated if (1) there is evidence that specific disease prevalence thresholds have been achieved and maintained for a minimum of two years in the absence of antibiotic MDA in each formerly-endemic evaluation unit (EU) of a country and (2) a suitable system is in place to identify and manage incident TT cases [3]. The elimination prevalence thresholds are a trachomatous inflammation—follicular (TF) prevalence <5% in children aged 1–9 years and a TT prevalence <0.2% amongst ≥15-year-olds [3].

Validation of elimination of trachoma as a public health problem does not require absence of ocular *Ct* infection [3–5]. Therefore, after antibiotic MDA has ceased, countries must monitor for potential recrudescence. However, there is no clear definition of trachoma recrudescence or re-emergence, including at what geographical level (community, sub-district or district) monitoring for it could or should be undertaken. While it may initially seem reasonable to define recrudescence as the transition of a district that had achieved a TF prevalence of <5% to subsequently having a TF prevalence ≥5%, TF prevalence is a lagging indicator as *Ct* prevalence declines [6–9] and could also conceivably lag behind *Ct* prevalence if infection subsequently recrudesced. Waiting until TF prevalence is ≥5% might therefore lead to missing opportunities for early intervention. Equally, the observation in some populations of moderate-to-high TF prevalence with low or absent ocular *Ct* infection raises a concern that other causes of follicular conjunctival inflammation might trigger an erroneous recrudescence alarm [10–14]. Either form of systematic misclassification (false negative or false positive) would be problematic for programmes.

The World Health Organization (WHO) guidance on methods for trachoma pre-validation surveillance has changed over time. In 2008, WHO recommended that surveillance for active trachoma be undertaken through examination of school-aged children for TF in 2–4 purposively selected sites per evaluation unit (EU). Sites in the “least developed and suspected most endemic” areas of the EU were to be selected [15]. In 2014, a WHO technical consultation [16] reviewed those recommendations and concluded that the methodology could be unduly influenced by bias and chance, compounded by an inadequate understanding of community-level factors that might be associated with *Ct* resurgence. The 2008 guidance was therefore replaced with a recommendation for pre-validation surveillance in each EU as a population-based surveillance survey carried out at least two years after the last impact survey [16]. There are no formal WHO guidelines regarding post-validation surveillance for active trachoma; this is a recommended activity of operational research, conducted within collaborations between health ministries and academic partners [3].

Designing operational research for post-validation surveillance is difficult because the evidence base for evaluation systems—when, where, who and what—is very limited. Several studies have examined use of serology and nucleic acid amplification tests (NAATs) as alternatives or adjuncts to TF for this purpose [4,17–22]. It may be possible to employ serology- and NAAT-based markers to identify communities at increased risk of recrudescence; this could begin pre- or post-validation [4], potentially in combination with surveillance activities for other diseases [23].

We present an evaluation of pre- and post-validation surveillance using serology and PCR, in the hope they can inform future refinement of trachoma programme surveillance strategies.

## Methods

### Ethical considerations

This research was approved by the Ghana Health Service (GHS) Ethical Review Committee (03/07/15 & 01/11/18) and the London School of Hygiene & Tropical Medicine Ethics Review Committee (reference: 10285 & 16169). Written informed consent was received from all participants and caregivers of minors, informed assent was also attained from children able to provide it. Individuals with TF were offered 1% tetracycline eye ointment. Individuals with TT were referred within the health system for management. This activity was reviewed by CDC and was conducted consistent with applicable federal law and CDC policy.

### Study area

This evaluation was conducted in two previously trachoma-endemic regions of Ghana: Northern and Upper West. In 2008, all districts had achieved the TF elimination prevalence threshold and antibiotic MDA was stopped [24]. In 2018, WHO validated Ghana as having eliminated trachoma as a public health problem [25,26].

### Study design

Two approaches for trachoma surveillance using clinical, antibody and infection indicators were evaluated to determine how effective they are in identifying on-going transmission and potential recrudescence.

1. Evaluation of the on-going pre-validation surveillance system, implemented by GHS from 2011–2015. This strategy was based on assessment of the prevalence of TF in children aged 1–9 years in a limited number of randomly selected sites each year. We will refer to these investigations as the **“TF trigger”** component of our study; it was done prior to validation, in 2015–2016.
2. Identification of communities hypothesized to be at potential risk of recrudescence on the basis of serological and infection data from either the TF trigger investigations or a series of pre-validation surveillance surveys conducted by GHS in 2015–2016 [25]. These communities were followed up in 2019. This work explored a potential model for post-validation surveillance. We will refer to the 2019 investigations as the “**infection and antibody trigger”** component.

**Fig 1:**
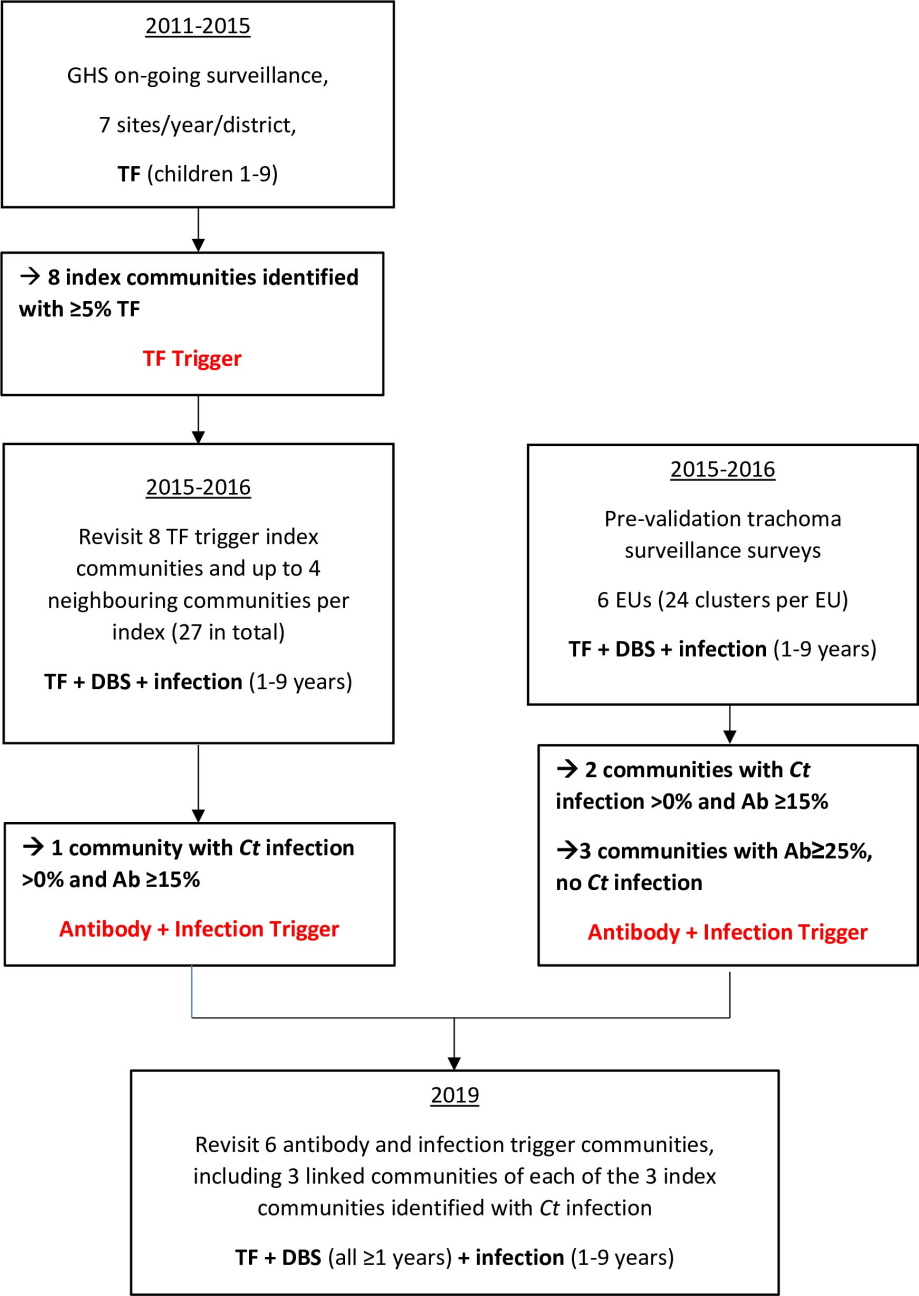
Flowchart depicting study methodology.

Fig 1 provides an overview of the study methodology and the various components.

### TF trigger evaluation

From 2011–2015, GHS implemented an on-going pre-validation surveillance system based on then-current WHO recommendations [27] consisting of field visits to seven randomly-selected sites per district per year in 18 EUs: two communities and five primary schools or pre-schools and the communities in which attending children lived. At those sites, all participants aged 1–9 years were examined for TF and all available participating adults aged ≥15 years were examined for TT. Once a school or community was visited as part of this process, it was removed from the sampling frame for all subsequent years. Through this methodology, from 2011–2015, a total of eight index communities were identified in which ≥5% of examined children had TF. GHS subsequently examined children for trachoma in up to four communities neighbouring those index communities; no further communities were found in which ≥5% of examined children had TF. Antibiotic MDA was undertaken in the eight index communities for three years [28]. The final MDA round was completed at least six months prior to the study investigations outlined below.

In 2015–2016, the eight index communities were each re-visited. For six of the eight communities, up to four neighbouring communities (identified by the health facility staff and based on proximity) were also visited. For the two districts (Sawla-Tuna-Kalba and Zabzugu-Tatale, in the northern region) in which more than one index community was identified, neighbouring communities were only chosen for the index community with the higher TF prevalence. In Sawla-Tuna-Kalba, the two index communities (Baale and Kema) are adjacent. All consenting individuals aged ≥1 year living in the index and neighbouring communities were examined for signs of trachoma and all children aged 1–9 years had a conjunctival swab and a dried blood spot (DBS) taken.

### Infection and antibody trigger

In 2015–2016, GHS conducted a number of population-based pre-validation surveillance surveys [25]; DBS and ocular swabs were collected from children aged 1–9 years in six of the EUs, as outlined in Senyonjo *et al*, 2018 [4]. The infection and antibody trigger method, took a step-wise approach, reviewing available *Ct* infection and anti-Pgp3 antibody data from either the pre-validation surveillance surveys or from the TF trigger investigations. This identified three communities as having, in 1–9-year-olds, (1) a prevalence of ocular *Ct* infection >0% and (2) a prevalence of anti-Pgp3 antibodies ≥15.0%. These three communities were included in the infection and antibody trigger investigations, with the expectation that they are the most at risk for potential recrudescence. For each of these communities, three communities “linked” to the index community were also invited to participate. The criteria considered for inclusion of a linked community were:

- Location within 5 km of the index community;
- Presence of a footpath or road directly linking it to the index community;
- Frequent interaction of residents with residents of the index community (based on report by residents of the index community), including (for example) through attendance of children at the same pre- or primary school.

Efforts were made to select linked communities from different compass points in relation to the index community.

Communities located in the same districts as the three index communities (Wa, Sawla-Tuna-Kalpa, Zabzugu-Tatale) that had a 2016 seroprevalence in 1–9-year-olds of ≥25.0% but no *Ct* infection were also invited to participate.

In February 2019, all consenting individuals aged ≥1 year in selected communities were examined for signs of trachoma and had a DBS taken. Children aged 1–9 years also had a conjunctival swab taken. A maximum of 300 DBS were collected per community, so in larger communities, children aged 1–9 years were exhaustively sampled and individuals aged ≥10 years were only sampled in every nth household, with n being the sampling interval calculated to result in the 300 DBS being collected evenly throughout the sampling frame. Data were also collected by interview on socio-economic status, access to water and sanitation services, education levels, school attendance, number of persons living in the household and compound (where applicable), and social interactions between children that could facilitate transmission. Efforts were made to include individuals who were initially absent, with teams returning a minimum of once to any household where one or more residents were not present at the time of the team’s first visit.

### Data handling

Survey data were collected electronically using secure, Open Data Kit-based Android smartphone applications (LINKS, Task Force for Global Health, Atlanta GA, USA, https://linkssystem.org, for the TF trigger investigations; CommCare, Dimagi Inc., Cambridge MA, USA, for the infection and antibody trigger investigations). Data were uploaded to a cloud-located server, which was password-protected with access only available to study investigators.

### Clinical assessment

Participating subjects were examined for the five signs of trachoma in the WHO simplified system: TF, trachomatous inflammation—intense (TI), trachomatous scarring (TS), TT and corneal opacity (CO)[29] by trained graders. Presence of pathology in one or both eyes was scored as that sign of trachoma.

All graders were ophthalmic nurses certified in 2015 using Global Trachoma Mapping Project methodologies [30,31], as previously described [25]. In 2019, graders were given refresher training on trachoma grading using photos; graders participated only if they achieved a kappa score of ≥0.8 for TF in an inter-grader agreement test.

### Sample collection

Swabs for *Ct* PCR were collected by passing a polyester-tipped swab three times across the upper tarsal conjunctiva of the left eyelid, using a standardised procedure described previously [4]. Precautions were taken to minimize field contamination of the swabs, including changing gloves after every sample taken. Negative control air swabs were taken by field teams after every 50 subjects for the TF trigger investigations and after every 25 subjects for the infection and antibody trigger investigations; the count restarted when the team moved to a new district. Samples were kept dry and cool in an ice box in the field.

To collect DBS, the participant’s finger was cleansed using an alcohol-soaked swab before being pricked using a sterile single-use spring-loaded lancet. Blood was collected directly onto a filter paper wheel with six projections, each calibrated to absorb 10 μl of blood. The filter paper wheel was air-dried in the shade before being individually packed in a sealable plastic bag and stored with desiccant.

Once collected, ocular swabs were stored at 4°C and transferred within a week on ice packs to Noguchi Memorial Institute for Medical Research (NMIMR), Accra, Ghana, where they were stored at -20°C until the time for analysis. The DBS were also stored at 4°C and (for the TF trigger investigations) transferred to NMIMR or (for the infection and antibody trigger investigations) sent to CDC, Atlanta, where they were stored at -20°C until being analysed.

### Detection of ocular *Ct* infection and anti-Pgp3 antibody

PCR for both sets of samples was conducted at NMIMR. For the TF trigger investigations, detection of anti-Pgp3 antibodies was conducted at NMIMR using an enzyme-linked immunosorbent assay (ELISA) as previously described [4]. For the infection and antibody trigger investigations, antibodies were detected using the multiplex bead array (MBA) platform at CDC, described elsewhere [32,33].

Conjunctival swabs were eluted using sterile diethylpirocarbonate (DEPC) water and pooled into groups of five samples using a published pooling strategy [34]. They were then analysed for the presence of *Ct* DNA using the GeneXpert CT/NG Assay run on GeneXpert IV (Cepheid, Sunnyvale CA, USA). For positive pools, component samples were re-tested individually to identify specific positive(s). Indeterminate pools were re-tested using a new aliquot of each component sample and a new GeneXpert cartridge. Field control swabs were analysed individually. Two *Ct*-positive and two *Ct*-negative processing controls were run at the beginning of each week.

### Statistical analysis

Statistical analyses were conducted using STATA (Version 17, StataCorp, College Station TX, USA) and R (R Core Team 2014). The threshold to determine a seropositive individual was calculated using a finite mixture model based on maximum likelihood methods, with a cut-off for positivity set as four standard deviations from the mean of the seronegative population [18]. For the MFI-bg values (data from the MBA) data were first log-transformed (log(x+1)), to accommodate zero values. For the samples analysed by ELISA, the cut-off was calculated as 1.091 OD_450nm_; for the samples analysed by MBA, the cut-off for log (MFI-bg+1) was calculated as 5.724.

Fisher’s exact test was used to assess the homogeneity of seroprevalence estimates between linked communities.

For the infection and antibody trigger investigations, simple associations between seropositivity in children aged 1–9 years and potential explanatory factors were evaluated using the Chi-square test or non-parametric test for trend, as applicable.

Seroconversion rates (SCR, the mean annual rate at which seronegative individuals become seropositive) were calculated using a simple reverse catalytic model (RCM) fitted to seroprevalence in one-year age groups (all ages ≥1 year), using maximum likelihood estimates [35]. Evidence for a change in SCR over time was conducted through comparisons of paired models, the first assuming constant transmission intensity, and the second assuming a change in transmission intensity at a specified time point [36].

For the infection and antibody trigger investigations, in communities in and around Mayido, spatial autocorrelation of household-level TF and seroprevalence (in children aged 1–9 years) within a village boundary was evaluated using Moran’s I index.

## Results

### TF trigger

A total of 1,869 children aged 1–9 years were present at the time of the survey and enumerated from across 27 communities (8 index, 19 neighbouring, Table 1). All were examined for signs of trachoma and had DBS taken. 1,831 DBS were analysed and able to be matched to demographic data. Of individuals matched to DBS results, 49.0% were female. 1,773 children had an ocular swab taken.

**Table 1:** Clinical, serology and conjunctival *C*. *trachomatis* (*Ct*) infection data from children aged 1–9 years in index and neighbouring communities visited as part of TF trigger-based investigations.

District	Community	TF prevalence 2011–2015*	Community type	n	TF prevalence in 2015–2016 pre-validation survey (n positive)	*Ct* infection (n positive)	Anti-Pgp3 antibodies (ELISA platform) (n positive)	Chi-squared test (Fisher exact) for seroprevalence (X^2^; p-value)
Sawla-Tuna-Kalba	Baale	6.8%	Index	56	0%	0%	16.1% (9)	15.8; 0.004
	Kema	6.7%	Index**	94	0%	0%	4.3% (4)	
	Soma		Neighbouring	140	0%	0%	2.1% (3)	
	Gbonbondouri		Neighbouring	119	0%	0%	9.2% (11)	
	Dani-uuri		Neighbouring	47	0%	0%	4.2% (2)	
West Gonja	Mampeasem	6.0%	Index	121	1.7% (2)	0%	2.5% (3)	6.1; 0.04
	Sakpege		Neighbouring	65	3.1% (2)	0%	0%	
	Lukula		Neighbouring	69	0%	0%	7.2% (5)	
Zabzugu-Tatale	Mangoase	7.8%	Index	58	1.7% (1)	0%	6.9% (4)	8.3; 0.03
	Jatoyili		Neighbouring	87	4.6% (4)	0%	1.2% (1)	
	Kpaligigbin		Neighbouring	44	0%	0%	13.6% (6)	
	Tasundo No 2		Neighbouring	62	0%	0%	6.5% (4)	
	Tamabug	6.1%	Index	60	1.7% (1)	0%	8.3% (5)	
Gushegu	Saguli	7.4%	Index	56	0%	0%	10.7% (6)	6.9; 0.11
	Galiwe		Neighbouring	60	3.3% (2)	0%	1.7% (1)	
	Tinyogu		Neighbouring	30	0%	0%	3.3% (1)	
	Zologu		Neighbouring	174	1.1% (2)	0%	3.5% (6)	
Wa	Kakalapere	6.6%	Index	74	5.4% (4)	1.4% (1)	16.2% (12)	7.0; 0.13
	Manliyiri		Neighbouring	14	0%	0%	28.6% (4)	
	Dariyiri		Neighbouring	43	2.3% (1)	0%	4.7% (2)	
	Bamkpama		Neighbouring	68	1.5% (1)	0%	11.8% (8)	
	Fooyiri		Neighbouring	14	0%	0%	7.1% (1)	
Nadowli	Moyiri	6.1%	Index	83	0%	0%	6.0% (5)	2.0; 0.68
	Nayikori		Neighbouring	89	3.4% (3)	0%	7.9% (7)	
	Wiche		Neighbouring	15	0%	0%	13.3% (2)	
	Dancheli		Neighbouring	55	0%	0%	7.3% (4)	
	Mwanwaare		Neighbouring	34	0%	0%	2.9% (1)	

*As per the methodology used by GHS at the time, the figure represents the highest prevalence reported either in the left or right eye

**Is in proximity to Baale and therefore is both an index and evaluated as a neighbouring community. Where there is more than one index community in a district, neighbouring communities are only selected for the index community with the highest TF prevalence.

TF: Trachomatous inflammation- follicular; *Ct*: *Chlamydia trachomatis*

**Fig 2:**
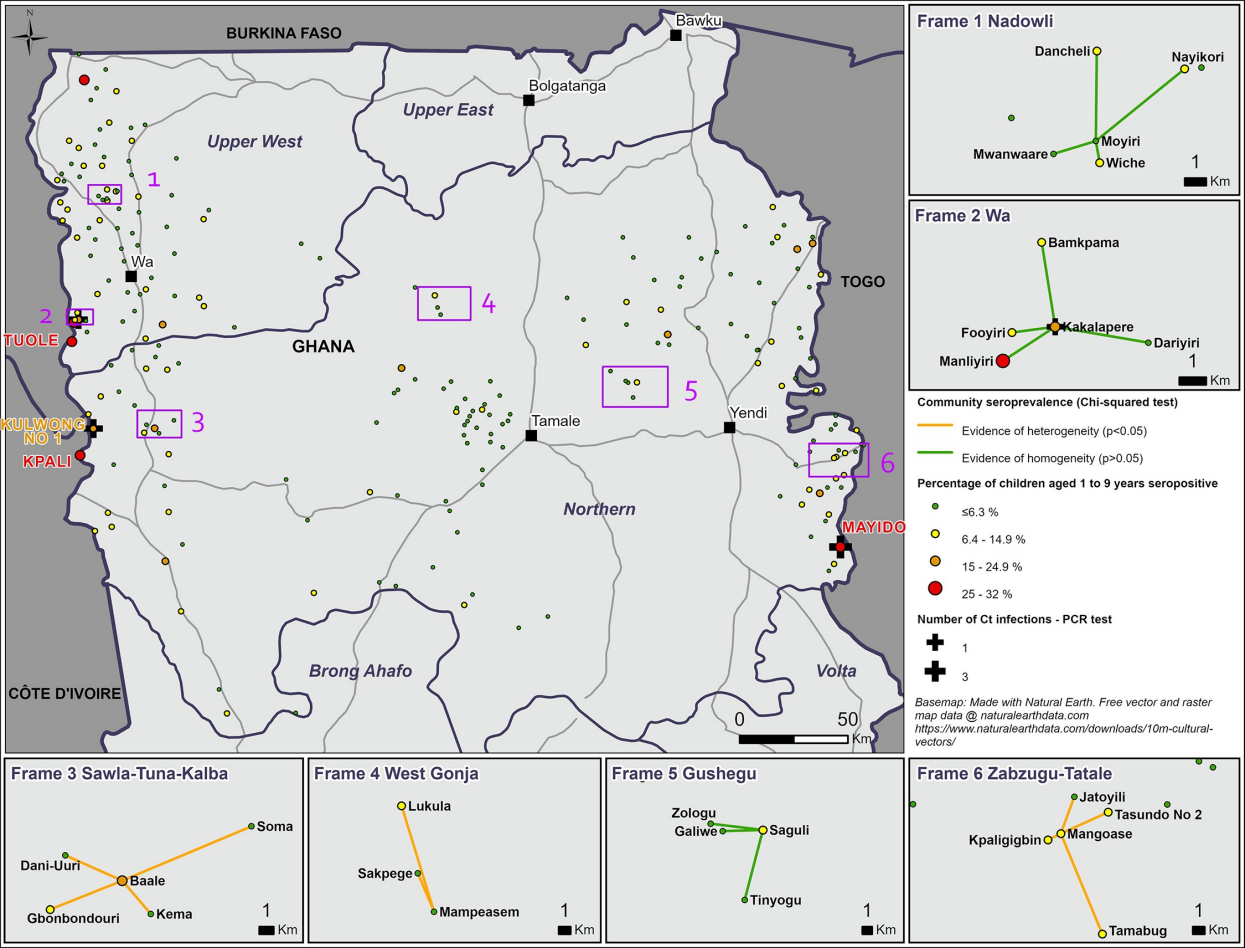
Map of seroprevalence by cluster, TF trigger investigations and pre-validation surveillance surveys.

Only one 1–9-year-old tested (in Kakalapere, Wa District) had a PCR-positive ocular swab. In this community, the *Ct* infection prevalence was 1.4%, the seroprevalence by ELISA was 16.2% and the TF prevalence was 5.4%, Table 1. The neighbouring community of Manliyiri had the highest seroprevalence recorded across the seven communities (28.6%). There was no evidence of a difference in the seroprevalence (p = 0.13) across sampled communities in Wa, Fig 2.

Data collected in 2015–2016. Base map: Made with Natural Earth. Free vector and raster map data @ naturalearthdata.com. https://www.naturalearthdata.com/downloads/10m-cultural-vectors/

There was an increase in seroprevalence by age, in Baale and neighbouring communities and likewise for Mampeasem and neighbouring communities, Table 2.

**Table 2:** Association between seropositivity and increasing age (1–9 years), TF trigger investigations.

District	Index (asterisks) and neighbouring communities	Non-parametric test for trend (z-score; p-value)
Sawla-Tuna-Kalba	Baale*, Kema*, Soma, Gbonbondouri, Dani-uuri	2.35; 0.02
West Gonja	Mampeasem*, Sakpege, Lukula	2.53; 0.01
Zabzugu-Tatale	Mangoase*, Jatoyili, Kpaligigbin, Tasundo No 2	1.36; 0.18
Gushegu	Saguli*, Galiwe,Tinyogu, Zologu	1.29; 0.20
Wa	Kakalapere*, Manliyiri, Dariyiri, Bamkpama, Fooyiri	1.56; 0.12
Nadowli	Moyiri*, Nayikori, Wiche, Dancheli, Mwanwaare	0.66; 0.51

### Infection and antibody trigger

The communities included were drawn from the TF trigger investigations described in the paper and the study published in Senyonjo et al, 2018 [37]. A total of 3,121 individuals were enumerated and present at the time of field teams’ visits; 52.7% were female. A total of 3,050 DBS were taken from individuals aged ≥1 year and 2,908 DBS were matched to demographic data. A total of 1,170 (37.5%) of those enumerated were aged 1–9 years, from whom 1,163 ocular swabs were taken and analysed.

One community (Mayido) had the highest prevalence of both *Ct* infection (1.6%) and anti-Pgp3 antibodies (14.4%) in 1–9-year-olds. Of the three cases of *Ct* infection identified in Mayido, two were from the same household. The community with the highest TF prevalence (Satinjado with 12.9% TF) was linked to Mayido, Table 3. Only one other community had any *Ct* infection—this community had only a single case of infection, no TF, and only 5.3% of children were positive for anti-Pgp3 antibodies, Table 3. There was no evidence of spatial autocorrelation of TF at household level in any of the communities in the district of Zabzugu-Tatale, where Mayido and Satinjado are located. Spatial autocorrelation of household seroprevalence was only identified in the community of Kogni (Moran’s I -0.157; p = 0.05), also in Zabzugu-Tatale.

**Table 3:** Clinical, serology and ocular *C*. *trachomatis* (*Ct*) infection data from children aged 1–9 years in index and linked communities visited as part of the infection and antibody trigger investigations.

District	Community	Community type*	n	TF prevalence (n positive)	*Ct* infection (n positive)	Anti-Pgp3 antibodies, MBA platform (n positive)	Chi-squared test (Fisher exact) for seroprevalence (X^2^; p-value)
Zabzugu- Tatale	Mayido (Kpaliba)	Index (*Ct* infection)	194	4.6% (9)	1.6% (3)	14.4% (28)	14.5; 0.004
	Satinjado	Linked	62	12.9% (8)	0%	3.2% (2)	
	Tilpado	Linked	127	6.3% (8)	0%	6.3% (8)	
	Kogni	Linked	149	4.7% (7)	0%	4.0% (6)	
Sawla-Tuna-Kalba	Kulwong no. 1	Index (*Ct* infection)	38	0%	2.6% (1)	5.3% (2)	0.4; 1.0
	Kulwong no. 2	Linked	38	7.9% (3)	0%	2.6% (1)	
	Chechereyili	Linked	36	0%	0%	2.8% (1)	
	Konhinyili	Linked	47	0%	0%	4.3% (2)	
	Kpali	Index (no infection)	79	2.5% (2)	0%	13.9% (11)	N/A
Wa	Kakalapere	Index (*Ct* infection)	92	0%	0%	3.3% (3)	3.0; 0.84
	Manliyiri	Index (no infection) & linked	17	0%	0%	0%	
	Deryiri	Linked	26	0%	0%	0%	
	Bamkpama	Linked	149	1.8% (8)	0%	3.4% (5)	
	Damwaateng	Linked	33	0%	0%	0%	
	Tuole	Index (no infection)	64	3.1% (2)	0%	6.3% (4)	N/A

*Ct infection denotes that the community had *Ct* infection and an anti-Pgp3 seroprevalence of >15% in 2016.

No infection denotes the community had no *Ct* infection identified but an anti-Pgp3 seroprevalence of greater than 25% in 2016 (amongst children aged 1–9 years)

There was strong evidence (p<0.001) of a change in the SCR at a time point between 6 and 15 years prior to sample collection in each of the three groups of communities evaluated. For the groups of communities in Sawla-Tuna-Kalba and Wa Districts, the more recent SCR (SCR2) was significantly lower than the historic rate after the time point of the rate change (SCR1). In Mayido and surrounding communities of Zabzugu-Tatale District, the more recent SCR was higher, Table 4.

**Table 4:** Seroconversion rates (SCR) within communities sampled, infection and antibody trigger investigations.

District	Index (and linked) communities	SCR 2 (most recent rate, after point of change)	SCR 1 (historical rate, before point of change)	Time point of change (years ago)	p-value
Zabzugu-Tatale	Mayido, (Satinjado, Tilpado, Kogni)	2.0 per 100 individuals per year (95%CI: 1.5–2.6)	0.4 per 100 individuals per year (95%CI: 0.3–0.7)	6	<0.001
Sawla-Tuna-Kalba	Kulwong no1, (Kulwong no2, Chechereyili, Konhinyili)	0.9 per 100 individuals per year (95%CI: 0.4–1.5)	5.8 per 100 individuals per year (95%CI: 3.7–9.1)	11	<0.001
Wa	Kakalapere, (Manliyiri, Deryiri, Bamkpama, Damwaateng)	0.6 per 100 individuals per year (95%CI: 0.4–0.9)	13.3 per 100 individuals per year (95%CI: 8.9–19.7)	15	<0.001

## Discussion

This study explored two approaches to identify recrudescence of trachoma. It provides evidence of heterogeneity in a post-validation setting, with variation in transmission dynamics, as might have been expected, over a relatively small spatial scale.

Our TF trigger methodology identified one community (Kakalapere) with a 2015–2016 TF prevalence ≥5%, anti-Pgp3 seroprevalence >15% and detectable ocular *Ct* infection and a neighbouring village with anti-pgp3 antibodies of >25%. When both communities were re-visited in 2019, there was no evidence of persistent infection or recent *Ct* transmission. The evaluation of serological indicators as part of the pre-validation surveillance surveys conducted in Ghana in 2015–2016, which provided evidence that the country had achieved the elimination thresholds, reported an overall cluster-summarised mean anti-Pgp3 seroprevalence of 5.5% (95% CI: 4.8–6.3) by ELISA. In and around Baale, in Sawla-Tuna-Kalba, there was evidence of an increase in seropositivity with an increase in age. Considering the community of Baale itself had a seroprevalence of over 15%, higher than the background seroprevalence rate, it could be argued that there is some evidence of past low-level transmission. However, this would be in line with the understanding of transmission dynamics in a post-validation setting and with no evidence of *Ct* infection, does not suggest recrudescence [37]. None of the other communities evaluated in the TF trigger work provided strong evidence of transmission within the previous nine years and none had current ocular *Ct* infection. While the TF trigger methodology was able to provide some evidence of limited *Ct* infection, it did not reveal significant onward transmission. A possible explanation for the lack of sustained transmission could be that there was transient infection in the community that was self-limiting, even if likely truncated as a result of the three additional rounds of MDA implemented prior to this study. Even so, the TF trigger approach likely lacks the spatio-temporal power to reliably identify communities experiencing recrudescence.

Evidence from the antibody and infection trigger data suggested that there has been persistent or repeated re-introduction of *Ct* infection over a three-year period in Mayido. While the *Ct* infection identified by serology could be ocular infection with urogenital *Ct* strains and therefore not indicative of trachoma, the convergence of indicators (infection, antibodies and disease) and household clustering of infections in two consecutive surveys suggests it was trachoma. The recent SCR increase seen in Mayido and its linked communities represents further evidence for on-going acquisition of anti-*Ct* antibodies and could be a warning sign of recrudescence. The trigger for programmatic action, whether that might be an absolute value or change in SCR alone, or one of those things in conjunction with exceeding a seroprevalence or infection threshold, still needs to be determined. It should be noted that observing an SCR increase here was only possible because we collected DBS from all ages, allowing for historical comparisons of transmission rates.

There are potentially other areas of northern Ghana with transmission dynamics similar to Mayido’s but that were missed as a result of the random sampling methodology that we used. It is therefore important to better understand the geospatial risk factors associated with on-going ocular *Ct* transmission in the post-elimination setting to guide programmes on where to focus resources for surveillance. We note that communities in and around Mayido differ from the surrounding population in having residents predominantly from the Konkomba ethnic group and mostly (66.7%) identifying as traditionalist. 2010 census data for Zabzugu district (split from Zabzugu-Tatale; containing the communities in question) [38] reported the main ethnic group of the district as Dagomba, with 36.0% of the population traditionalist. Further work will be required to understand spatial connectivity and the contextual factors that lead to re-introduction and facilitation of on-going *Ct* transmission between individuals [39]. Differences in social and behavioural practices, especially related to hygiene and sanitation, would be interesting to explore further.

It has been suggested that the distribution of infection in a peri-elimination setting would best fit an exponential curve, which has a heavy tail, and that therefore communities with higher infection prevalence would be occasionally expected. The implicit corollary is that community prevalence would regress to the mean over time [40]. Regression to the mean has been observed in hypo-endemic settings in Nepal, Tanzania and the Gambia, where infection disappeared in relatively-high-prevalence communities [8,41–44]. The present study demonstrates that this phenomenon can be either delayed (beyond 10 years) or even fail to occur. We identified an area in which *Ct* infection persisted, with transmission potentially increasing post-validation, many years after district-level MDA was stopped. Interruption of *Ct* transmission does not quickly and invariably follow attainment of the criteria for trachoma’s elimination as a public health problem. If the goal of transmission interruption is to be proposed [45] it should be noted that it may not occur without additional measures to identify and address such foci.

Some of the limitations of this study relate to the interpretation of test results. Infection and antibody tests do not distinguish ocular from urogenital *Ct* infection, and TF can arise from other local inflammatory stimuli [46,47]. The reverse catalytic model to calculate SCR may be an over-simplification of real-world transmission dynamics and estimates could be improved by using a model based on antibody levels and multiple time points [48]. There are also difficulties in evaluating community prevalence indicators in small communities, such as Manliyiri, which reported a large swing in seroprevalence from 28.6% in 2015 to 0% in 2019. Although every effort was made to reach all children aged 1–9 years, the omission of only a few children that may have been seropositive or the graduation (in age) of seropositive children over the time period, shifting them outside the sampling frame in 2019, would have a large impact on overall prevalence estimates.

Evidence of transmission between nearby communities could have important implications for wider recrudescence that may be of concern to programmes. The situation in and around Mayido is difficult to interpret, as although TF was identified across all four communities, there was varying anti-Pgp3 seroprevalence, ranging from 3.2% to 14.4% and generally low levels of *Ct* infection. There may have been low-prevalence *Ct* infection but it was not identified either because of the sensitivity of the test, the pooling process used for sample analysis or issues with swab collection or transport. It would be worthwhile to revisit this area after a minimum of two years, to evaluate if there is any evidence of an increase in ocular *Ct* transmission mandating programmatic intervention.

## Conclusion

There is evidence of heterogeneity in ocular *Ct* transmission dynamics after validation of trachoma elimination as a public health problem, even 10 years after wide-scale MDA has stopped. There is no suggestion from this study of a threat to elimination thresholds in Ghana. Elimination thresholds for trachoma are evaluated at the level of the district or administrative unit with a population of 100,000–250,000. This study has identified on-going transmission at a smaller geographic scale and the importance of and programmatic implications of such foci are still to be determined. However, such foci should be routinely observed to monitor potential recrudescence over a larger geographic scale. There is added value in monitoring anti-*Ct* antibodies and *Ct* infection, either within routine surveillance or to interrogate past strategies. This can result in a deeper understanding of transmission dynamics and inform new post-validation surveillance strategies. Opportunities should be explored for integrating infection and serology-based markers into other infectious disease surveillance surveys conducted in trachoma elimination settings.
